# A237 INCIDENCE, RISK FACTORS, AND OUTCOMES OF POST-ERCP CHOLANGITIS: AN INTERNATIONAL MULTICENTER PROSPECTIVE STUDY

**DOI:** 10.1093/jcag/gwae059.237

**Published:** 2025-02-10

**Authors:** M Gupta, M Fazal, J Hammal, S Ficaccio, R Khan, N Forbes

**Affiliations:** University of Calgary Cumming School of Medicine, Calgary, AB, Canada; University of Alberta Faculty of Medicine & Dentistry, Edmonton, AB, Canada; Division of Gastroenterology and Hepatology, University of Calgary, Calgary, AB, Canada; Division of Gastroenterology and Hepatology, University of Calgary, Calgary, AB, Canada; Division of Gastroenterology, Mayo Clinic Minnesota, Rochester, MN; Division of Gastroenterology and Hepatology, University of Calgary, Calgary, AB, Canada

## Abstract

**Background:**

Cholangitis is a well-described adverse event (AE) following endoscopic retrograde cholangiopancreatography (ERCP) that is associated with significant morbidity and mortality. Despite this, contemporary characterization of this AE using high-quality prospective data is incomplete.

**Aims:**

In this study we describe post-ERCP cholangitis incidence, risk factors, and outcomes in a large, international multicenter population of patients undergoing ERCP.

**Methods:**

We analyzed prospective data from nine centers in Canada, USA, and Europe between 2018-2024 with 30-day follow-up. The primary outcome was post-ERCP cholangitis, defined as (1) temperature <36.0°C or >38.0°C or white blood cells <4 x10^9^/L or >10x10^9^/L, (2) accompanying rise in bilirubin or transaminases to >1.5 times the upper limit of normal or any increase compared to pre-procedure, *and* (3) emergency department visit or hospital admission and/or prolongation of existing admission. Multivariable logistic regression was performed to identify risk factors, with results presented as odds ratios (OR) with associated 95% confidence intervals. Outcomes following cholangitis were also described.

**Results:**

Post-ERCP cholangitis occurred following 134 (1.7%) of 8,119 ERCPs (mean onset of symptoms 2.0 days). Repeat ERCP was required in 53.7% of these cases. Mortality attributed to cholangitis occurred in 8.2% of incident cases. Male sex (OR 1.48, 1.02-2.19), longer procedural time (OR 1.01, 1.00-1.02 per additional minute) and presence of a biliary stricture (OR 3.24, 1.98-5.30) were all independent risk factors for post-ERCP cholangitis, while placement of a biliary stent (OR 0.59, 0.36-0.95), and biliary sphincterotomy (OR 0.59 0.39-0.87) were both protective against post-ERCP cholangitis.

**Conclusions:**

Post-ERCP cholangitis is common and associated with high mortality rates. Adequate pre-procedural counseling and early recognition is advised, especially in patients at increased risk.

**Funding Agencies:**

None

